# Dreams and Visions

**Published:** 1891-01

**Authors:** N. S. Hoff

**Affiliations:** Ann Arbor, Mich.


### "Your Old Men Shall Dream Dreams, and Your Young Men Shall See Visions."

BY N. S. HOFF, D.D S., ANN ARBOR, MICH.

Read before the Ohio State Dental Society, held at Columbus, October, 1890.

In the January number of the *Dental Review* appeared a vision, given to one of our younger men, of the conditions under which dental science and its professional practice will continue to alleviate the sufferings, and enlighten the conscience of mankind ten years hence. Dental education, journalism, commercial relations, patent laws, etc., will all undergo a process of change that shall bring them into more harmonious relations.

Another dreamer, a member of the Southern Dental Association, fancies a permanent home for that association, located on the top of one of the high mountains of Georgia, Tennessee or North Carolina, where the pure air will prove an alluring invitation to the weary practitioner, and where he may go and lay aside the vexations and burdens of his daily avocation to enjoy, not only the pure air and wholesome food, but can have the privilege of pursuing any scientific investigation dear to his heart, with the aid of a complete library, museum, and experimental laboratory.

At the last meeting of the American Dental Association the president of the association, and the chairman of the section on Dental Education, also related visions of a uniform dental law regulating the practice of dentistry in all States, the establishment at Washington of a dental museum and library, and the organization of a national university with power to confer a special degree for superior scientific attainment.

Then there is the dream that so constantly and tenaciously occupies the great mind of our vigorous and emphatic western friend who is looking forward anxiously to the time when he shall free the dental profession of all avaricious patent-right speculators, and after buying all legitimate patents, hold them for the use of members of the Dental Protective Association.

But the latest, and one of the best, comes from the east in the announcement that a prominent member of the profession in New York City has generously offered to contribute five thousand dollars toward the establishment of a dental club. Not as a convenient place for idling away precious time over a game table, or in idle conversation with its usual pernicious accompaniment; but this idea contemplates a commodious building with apartments containing a dental museum, library, and laboratory fully equipped for carrying on investigations in all kinds of scientific work that has a bearing on dental practice. The plan also contemplates rooms for holding meetings of dental societies, and for giving clinics.

The consummation of this design will give a greater stimulus to the tendency and hope of dentists to become truly scientific men, and to place their profession on a scientific basis than all the discussions ever held on the subject of dental education.

But speaking of dreams, let me outline another, not the result of a stomach overloaded with indigestible sweet-meats, but probably traceable to the above outlined visions of the wise men of the east, south, and west.

If it is good for New York, Atlanta, and Washington to provide homes for their respective society gatherings, and places for professional culture, why should not the great State of Ohio with its wealth of men and brains have a place of assembly at some central and accessible point providing facilities and accommodations of library, museum, and laboratory for advanced work in all branches of professional attainment ? No argument need be made to illustrate the benefit of such a provision, and none is necessary to demonstrate conclusively the demand for such an institution, certainly not to those that are connected with dental colleges where almost every mail brings a letter of inquiry from some practitioner as to the facilities afforded for post-gruduate work in various lines of scientific investigation.

Now that the Ohio State Society has returned to Columbus for another meeting is it not a fitting time and place to discuss this subject and determine whether some plan can not be devised which shall accomplish this object? Would not the city of Columbus, with its central location, its capital and city advantages, furnish an acceptable place for locating a permanent home of this character for the State Society ? If there is no Bodecker in Ohio to inaugurate this enterprise, there is nothing to prevent the organization of local societies in every county or large city (with the idea of increasing the membership of the State Society by making each local society auxilliary to it), and extending to the members of such societies all the privileges of the State Society including the use of library and laboratories, the State Society exacting a *per capita* assessment from all such societies to be used in supporting and carrying on its work. If it is impracticable to purchase property and erect a suitable building, the collection of the library and museum could be commenced at once and a building fund could be started. In the mean time such a building or rooms as are needed could be rented, and the work practically organized. If this were done and a competent man to put in charge could be found, who would be willing to undertake the work at a small salary, and devote his entire time and energy to the organization of local or auxilliary societies; collecting and tabulating his work, and publishing the same in a journal conducted by the society; assisting and counseling post-graduate students, and original investigators; arranging with specialists for courses of lectures at stated intervals to classes of post-graduate students; collecting and classifying the museum and library, and making it available for investigators; and with the assistance of a committee appointed for the purpose a programme could be made out embracing suitable work to be done by each auxilliary society, and once each year the whole compiled into a report to be read and discussed at the annual meeting, taking the place of the heterogeneous programmes usually presented at society meetings. If this were done the profession in one State, at least, would be doing for its members a work that would bring great honor to it, and the latent talent now peacefully reposing in its members would be aroused, and Ohio would produce more Atkinsons and Millers. There is no great difficulty in inaugurating some such plan on the plea of expense. The fees from post-graduate students would pay the rent of the rooms and assist in furnishing them, and there are certainly 1,000 or 1,500 dentists in and out of the State who would subscribe to a good live dental journal where they know that all its profits go to the support of such an institution, and this, with the annual dues of members would furnish all needed income. There are fragments here and there in the libralies and closets of dental practitioners that would soon find their way into the State library and museum. By such an undertaking the society would commend itself to the consideration of professional millionaires, and gifts of money would fall to it, and possibly some recognition would be taken by the State Government of a substantial character. And so by gradual accumulation the rolling stone would gather sufficient moss for the erection of a substantial habitation.

